# Supplementary material for: The Daily Mile: teachers’ perspectives of the barriers and facilitators to the delivery of a school-based physical activity intervention
Source: BMJ Open. 2019 Mar 4;9(3):e027169. doi: 10.1136/bmjopen-2018-027169 (PMC6429867; doi:10.1136/bmjopen-2018-027169)
Supplement: Supplementary file 1 [file bmjopen-2018-027169supp001.pdf]

Appendix 1. Daily Mile Interview Topic Guide

Does the Daily Mile take place at this school? If so, can you give us a brief history of its use at the school? (Were you given any assistance/guidance when setting it up?)

Can you describe a typical day at school for your class- when does the Daily Mile come into play?

What is your understanding of what the Daily Mile actually is? (what does it involve, what are its objectives etc)

When it was first introduced, how did you feel about it? (and have your feelings changed since its implementation?)

Is physical activity a major focus at this school? *(Probe for any physical activity policies that are in place, what does physical education involve/how often is it, is the playground appropriate for physical activity).*

Do you think the children that you teach should be more physically active? *(Are there noticeable issues related to physical activity in the class, obesity, generally unfit etc.).*

In general, how well are the children you teach performing academically? *(are there many children with additional support needs?*

Are there any behavioural issues in the class? *(How well do they listen and follow instructions? Are there any particular times of the day when they are more disruptive/easier or harder to teach?)*

Does their behaviour change following the Daily Mile at all?

Are there any wider issues within the community in which this school is located that you think may hinder/enhance the achievement of children at school? *(Look to discuss the deprivation/affluence of the area, level of engagement with children's learning and development from parents, area safety and crime, provision of adequate facilities. Do these same issues, or others, possibly impact on the children's physical activity levels?).*

Considering all the things we have just discussed, what effect do you think the Daily Mile programme would have on children at this school? *(How would it effect their physical activity levels, would this benefit their overall health? Do you think it would have any effects on their learning or behaviour in class?).*

Thinking practically about what the Daily Mile programme would actually involve, how feasible was it to implement the Daily Mile programme at this school, and keep it running on a daily basis? *(Do you think it is worth sacrificing the classroom time to do it? Do you think other teachers at this school would be in favour of doing the Daily Mile with their class?).*

Were you aware of any challenges that came to light as the Daily Mile was being implemented?

What have been the main barriers for implementing this programme here?

The first school to use the Daily Mile has an all-weather track around the playground for children to use. Do you think the playground at this school is adequate for the Daily Mile to

take place? *(Is there enough space? Would you need to build an all-weather track? What other resources do you think would be required to implement and sustain the programme here?)*

What specific benefits do you think the Daily Mile is having on children at this school? *(Health benefits, academic benefits, behavioural benefits?)*

How do you think parents feel about their children taking part in the Daily Mile, are they aware of it?

What do you think the children think of the Daily Mile?

Considering everything we have discussed, and the specific issues that are present in this area and at this school do you think the introduction of the Daily Mile has been a positive thing overall?

What has been the response from your colleagues?
