# Supplementary material for: The Daily Mile: teachers’ perspectives of the barriers and facilitators to the delivery of a school-based physical activity intervention
Source: BMJ Open. 2019 Mar 4;9(3):e027169. doi: 10.1136/bmjopen-2018-027169 (PMC6429867; doi:10.1136/bmjopen-2018-027169)
Supplement: Author's manuscript [file bmjopen-2018-027169.draft_revisions.pdf]

BMJ Open is committed to open peer review. As part of this commitment we make the peer review history of every article we publish publicly available.

When an article is published we post the peer reviewers' comments and the authors' responses online. We also post the versions of the paper that were used during peer review. These are the versions that the peer review comments apply to.

The versions of the paper that follow are the versions that were submitted during the peer review process. They are not the versions of record or the final published versions. They should not be cited or distributed as the published version of this manuscript.

BMJ Open is an open access journal and the full, final, typeset and author-corrected version of record of the manuscript is available on our site with no access controls, subscription charges or pay-per-view fees (http://bmjopen.bmj.com).

If you have any questions on BMJ Open's open peer review process please email info.bmjopen@bmj.com

# The Daily Mile: Teachers' perspectives of the barriers and facilitators to the delivery of a school-based physical activity intervention


| | |
|---|---|
| Journal: | *BMJ Open* |
| Manuscript ID | bmjopen-2018-027169 |
| Article Type: | Research |
| Date Submitted by the Author: | 17-Oct-2018 |


| | |
|---|---|
| Complete List of Authors: | Malden, Stephen; University of Edinburgh, Scottish Collaboration for Public Health Research and Policy, School of Health in Social Science, Doorway 6, Old Medical School, Teviot Place, University of Edinburgh, Edinburgh ; University of Strathclyde School of Psychological Sciences and Health, Physical Activity for Health Group<br>Doi, Lawrence; University of Edinburgh, Scottish Collaboration for Public Health Research and Policy, School of Health in Social Science, Doorway 6, Old Medical School, Teviot Place, University of Edinburgh |


| | |
|---|---|
| Keywords: | physical activity, daily mile, education, teacher, school |


SCHOLARONE™
Manuscripts

# The Daily Mile: Teachers' perspectives of the barriers and facilitators to the delivery of a school-based physical activity intervention

Stephen Malden[1,2] stephen.malden@strath.ac.uk,

Lawrence Doi[1] larry.doi@ed.ac.uk

[1]Scottish Collaboration for Public Health Research and Policy, School of Health in Social Science, Doorway 6, Old Medical School, Teviot Place, University of Edinburgh, Edinburgh EH8 9AG, UK

[2]Physical Activity for Health Group, School of Psychological Sciences and Health, Graham Hills Building, room 535, 40 George Street, University of Strathclyde, Glasgow G1 1QE, UK

Corresponding author: Stephen Malden

Word count: 5334 words

For peer review only

## Abstract

**Objectives:** Children spend a significant amount of their time in a school environment, often engaged in sedentary activities. The Daily Mile is a physical activity intervention, which aims to increase physical activity and fitness in children through the completion of an outdoor teacher-led walk or run during the school day. This study aimed to explore the barriers, facilitators and perceived benefits of the Daily Mile from the perspectives of teachers through the use of qualitative semi-structured interviews. It also aimed to identify important context-specific factors, which might require consideration for those who intend to adopt the Daily Mile.

**Setting:** Eight local authority primary schools in the City of Edinburgh and East Lothian, UK.

**Participants:** Thirteen teachers (ten women) who teach children in primaries one to seven in a school which delivers the Daily Mile.

**Results:** Data were analysed using an interpretative thematic analysis. Teachers were positive and enthusiastic about the Daily Mile and perceived it to be beneficial to children's health and fitness. A number of barriers to participation were identified including inadequate all-weather running surfaces and time constraints in an already full school curriculum. The perceived impact on learning time was identified as a concern for teachers, while other benefits were also identified including increased teacher-child rapport and perceived enhanced classroom concentration levels.

**Conclusion:** The Daily Mile appears to be a valuable addition to the school day, however important context-specific barriers to delivery of the Daily Mile exist which should be considered when implementing the Daily Mile in schools.


**Keywords:** Physical activity, daily mile, education, teacher, school



For peer review only - http://bmjopen.bmj.com/site/about/guidelines.xhtml

**Strengths and limitations of the study**

- One of the first studies to investigate teachers views of the daily mile
- Sampled teachers from schools in multiple locations to investigate contextual factors
- Data collection with children wold have allowed for data triangulation
- Small sample size may limit generalisability of findings

**Introduction**

A lack of physical activity is a determinant of numerous health conditions in childhood [1]. Specifically, sedentary behaviours and low levels of physical activity are partly to blame for the marked increases in childhood obesity that have been observed internationally in recent years [2 3]. In the United Kingdom, childhood overweight and obesity levels are high, approximately 30% [4]. Additionally, physical activity levels are low [5] and have been shown to decrease with age, a trend that has been similarly observed in other European countries and the United States [6]. Considering this, there is a need for the implementation of effective, evidence-based interventions to increase physical activity levels in school-aged children.

The Daily Mile is an example of a school-based intervention that involves teachers taking their class outside at some point during the school day to participate in 15 minutes of exercise. Children choose what pace they complete the activity, with those who run for the full 15 minutes likely to have completed approximately one mile. The intervention is intended to be delivered in addition to the 2 hours per week of quality physical education as required by the Scottish Curriculum [7], and is not intended to replace this time. The Daily Mile was originally introduced to primary schools to increase fitness levels [8], but it may also have a positive effect on physical activity, sedentary behaviour and body composition [9]. Since its inception at St Ninian's primary


For peer review only - http://bmjopen.bmj.com/site/about/guidelines.xhtml

school in Stirling in 2012, participation and expansion of the initiative has grown exponentially, with the Daily Mile now taking place in more primary schools across Scotland, in addition to a number of sites both within the UK and internationally [8].

A recent quasi-experimental pilot study by Chesham et al. (2018) showed that when compared to control schools, children who participated in the Daily Mile  showed significant improvements in objectively-measured daily moderate-vigorous physical activity (MVPA), objectively-measured sedentary time, fitness and body composition [9].  To date, the aforementioned study is the only research currently offering empirical evidence of the Daily Mile's efficacy, although another randomised trial is being planned [10]. However, interventions do not usually work the same everywhere, and it is important to understand contextual factors that may influence how they work [11]. Currently very little is known about factors that may influence the efficacy of the Daily Mile intervention. Teachers play a crucial role in terms of delivering the Daily Mile and it is important to understand their perspectives in order to inform learning, especially for schools and policy makers who intend to adopt the intervention. Contextual issues have been found useful in other school programmes, where context-specific differences have been considered when testing and developing an intervention [12]. Therefore, examining the experiences and perspectives of teachers in early adopter schools can allow policy makers and practitioners to adapt the Daily Mile to suit their settings where necessary. A large number of teachers are now delivering the Daily Mile, however at present there appears to be no published study, which has highlighted teachers' experiences and perspectives of the Daily Mile and their perceived impacts on their practice. This study explored the barriers, facilitators and perceived benefits of the Daily Mile from the perspectives of teachers who deliver the intervention. It also aimed to identify important



For peer review only - http://bmjopen.bmj.com/site/about/guidelines.xhtml

context-specific factors, which might require consideration for those who intend to adopt the Daily Mile in the future.

## Methods

### *Patient and Public Involvement*

A senior member of teaching staff (physical education coordinator) who had well established contacts with teachers in multiple schools acted as a gatekeeper during recruitment and liaised with potential participants. The research findings will be shared with the participants in a short summary report which will be emailed to them directly and to head-teachers of the participating schools.

### *Design*

Adopting interpretive approach, qualitative methods were used to explore teachers' perspectives of the Daily Mile and their experiences of delivering it. Semi-structured interviews were conducted, consisting of open-ended questions which aimed to determine how teachers define the Daily Mile, how they deliver the initiative, perceived benefits for the children who participate, and potential context-specific barriers and facilitators to participation. Our goal was to explore their experiences of the Daily Mile in order to inform future practice rather than to develop new theory.

Ethical approval was granted by The University of Edinburgh's Usher Research Ethics Group, and Local Authority research in schools permission was obtained prior to recruitment. All

For peer review only

participants were given an information sheet at the start of the study, before providing written informed consent.

***Setting, participants and recruitment***

This study was conducted in Local Authority primary schools within two council areas: the City of Edinburgh and East Lothian, Scotland. The sites contain significant rural and urban populations, while also having both some of the most affluent areas in the country and some of the most deprived areas, as defined by the Scottish Index of Multiple Deprivation (SIMD) in Table 1 [13].

Primary school teachers who teach primaries one through seven at a school, which participated in the Daily Mile or had done so in the past were recruited from the participating schools. As it is mostly class teachers who deliver the Daily Mile, head-teachers, or teaching staff who do not have regular prolonged contact with children were excluded from participating. A total of thirteen teachers took part in the study, consisting of eleven women and two men.

A purposive sampling strategy was used to recruit teachers. Specifically, contact was made with a physical education coordinator in one of the Local Authority areas who acted as a "gatekeeper". Between August 2016 and February 2017, the gatekeeper delivered study information sheets and personally spoke to selected head-teachers about the study. In order to enhance participants' recruitment, emails and letters were sent to head teachers of additional primary schools. This contained study information sheets and researcher's contact details. Twenty-one schools were approached to participate in the study, for which ten initially agreed to participate. Teachers from eight of the ten schools subsequently agreed to participate in the study.

For peer review only

***Data collection***

Semi-structured interviews were conducted face-to-face in a private room within the participating schools by one researcher. Participants were asked to give a brief history of the Daily Mile at their school, before probing questions were asked regarding subjects such as how the initiative is viewed by the staff at the school, approaches taken to deliver the program, and any perceived benefits, facilitators, or barriers associated with participation and delivery. A sample of the interview topic guide can be viewed in appendix 1. Interviews were usually conducted after the school day and lasted between 21 and 58 minutes.

***Data analysis***

Utilising interpretive approach, this study employed thematic analysis to analyse the qualitative data that were generated following verbatim data transcription. Firstly, a coding framework was developed by both authors who independently coded the same two transcripts from the dataset, before collaborating to refine the coding framework. This was then applied to all remaining transcripts. Similar codes were grouped together into overarching themes. The analysis proceeded to the interpretive phase where similarities and differences between units of the data underwent dynamic series of negotiations before patterns of meaning and understanding developed. Data analysis was conducted using NVivo 10 qualitative data analysis software.

**Results**

Characteristics of schools

For peer review only

The characteristics of the eight schools where the thirteen teachers were drawn from varied with regards to facilities, rurality and level of deprivation as detailed in Table 1. All but one school had access to sufficient greenspace within the school grounds, while half of the schools had access to an all-weather surface such as an Astroturf football pitch or a solid polyurethane running track. Three schools were located within a large town or city, three were in a semi-rural location such as a small country town, and two schools were in a rural location. Three schools were located within areas of high deprivation, three within areas of medium deprivation and two in affluent areas.

Table 1. Characteristics of schools

| School Identifier | Accessible greenspace in school grounds | Accessible all-weather surface in school grounds (such as Astroturf) | Rurality (rural, semi-rural, urban) | Level of deprivation (SIMD quintile, 1= most deprived 5= least deprived) |
|---|---|---|---|---|
| A | Yes | No | Semi-rural | 3 |
| B | Yes | Yes | Semi-rural | 5 |

For peer review only

| | | | | |
|---|---|---|---|---|
| C | Yes | Yes | Urban | 3 |
| D | Yes | No | Rural | 4 |
| E | Yes | No | Urban | 1 |
| F | No | Yes | Urban | 1 |
| G | Yes | No | Rural | 5 |
| H | Yes | Yes | Semi-rural | 1 |

**Themes**

Eight themes were identified, and detailed below.

***Style of delivery***

The style of delivery varied considerably between teachers and schools. While each school followed a similar Daily Mile protocol for delivery, some schools offered more autonomy to teachers concerning how and when they delivered the Daily Mile.

> *So yeah, it does happen in this school and what we do is that we split it, we don't do the mile in one go, we do half a mile in the morning and half a mile in the afternoon and it's immediately after break, or immediately after lunch, with the exception of Thursdays when it really works in our favour because we do it immediately after assembly. Because the kids have been sitting down and just not, being in a stuffy hall for, you know, half an hour.* F2

There were also some differences in the frequency and level of engagement with the Daily Mile amongst the schools. A number of participants stated that all classes at their school took part,

For peer review only

often at the request of the head teacher, while others mentioned that only some classes did the Daily Mile, and that participation does not happen every day either due to teacher preferences or age/ability of the children.

> *Some of the other classes do it every day as well, but some just do it every now and then. F1*

> *I know that the [primary] ones certainly don't do it because it's a lot more difficult to manage.* B2

There were slight variations in how the Daily Mile was defined by the teachers. Variations in what constitutes participation were evident, with some teachers defining the Daily Mile as 15 minutes of exercise, and others as an exact mile that is done to completion regardless of time taken. The emphasis on running the Daily Mile also differed between teachers and schools, with some teachers' strongly encouraging running, while others were happy for children to choose whether to walk or run.

> *I don't start by saying, you can run or walk it, I start by saying, it's a run and I sneakily tell people they can walk if I think that, you know, they need to. But even if you tell them to walk, quite often they end up running*. D2

The time at which the Daily Mile was delivered during the school day also differed amongst the teachers. Some participants stated that they purposefully delivered the intervention at set times during the day in order to establish a routine or get a desired behavioural or performance-based response from the children.

For peer review only

*I'm doing it after break and lunch. I think that's really effective because you don't need to take them out. There's no difference. They're already outside. They can take their jackets off or they can leave them on.* F1

*I've done it in the middle of the afternoon rather than straight after break – I much prefer that. I prefer to use it as a break in the lessons or change of lesson.* D1

There was variability amongst the sample regarding the interaction between the Daily Mile, and formal physical education sessions. A number of teachers explicitly stated that they do the Daily Mile in addition to the 2 hours per week of physical education recommended by the school curriculum. However, a few of the teachers asserted that they count the Daily Mile as part of the physical education curriculum and therefore as a part of the 2 hours of weekly PE which they are expected to deliver.

*See, I didn't know that [the Daily Mile does not count towards PE time]. I've sometimes done it as a warm-up, so I've done the Daily Mile and then 45 minutes of PE and then don't have to do a warm up in PE.* D1

*I think people were concerned about where would it fit, in the timetable, because obviously we're stretched for our two hours of PE, and now we have to do the Daily Mile. Last year I did it as part of my PE.* B2

***Motivating children to participate***

Approximately half of the teachers used some form of motivational or incentive/rewards-based system with children when doing the Daily Mile. Such techniques included the handing out of tokens for each lap of the running area completed, or tracking the distance ran over an accumulative time-period and matching it with the distances to various geographical locations.


For peer review only - http://bmjopen.bmj.com/site/about/guidelines.xhtml

*With the Primary 1s and 2s and 3s last year, we gave them…you know the Unifix Cubes that stick together? So every time they complete a lap, they get a cube. And they add them on. And it was good in so far as we could use that then as a numeracy lesson and obviously for the little ones, you know, they were counting, they were being inspired.* D1

*We have a big board at the front of the school and it shows everybody's progress tracked. We are aiming to go to different places in the world and to the moon as a whole school. It's really positive and they are motivated.* H1

Some teachers stated that they found that their own participation in the Daily Mile motivated the children to try to keep up or beat them, thus increasing the children's engagement with the activity.

*Every now and again I'll go and run with them. But they'll always, you always see a boost in them when, "the teacher's coming, the teacher's coming, he's coming!" And then I'll just go, oh come on guys, I'm beating you and I'm so much older. And it just adds something to it.* F2

***Health, wellbeing and fitness***

A number for the teachers attributed the Daily Mile to improvements in general health and fitness that they observe amongst children in their care. Specifically, the majority of the teachers indicated that the children's time taken to complete the Daily Mile had generally improved since the programme started, and others attributed school success in sporting events to Daily Mile participation



For peer review only - http://bmjopen.bmj.com/site/about/guidelines.xhtml

*We interviewed some of the children about how they'd got on [competing in an athletics competition]. Actually their reflections were, we think we did better because we are fitter because we do the mile and that wasn't prompted at all, so I think that they feel fitter and healthier.* C1

*They are definitely fitter because I forgot to say that we are recording last year's Primary 5s at the start of the year and we did fitness levels with them before we even started the mile. We've done the same cohort of children every term since and we have that recorded as well for every single child's results we can see that they are all fitter, so that's pretty good.* H1

Two teachers also speculated that Daily Mile participation led to improvements in asthma symptoms amongst some children. One of the teachers explained this below.

*I'm seeing a great difference in kids with asthma. Huge. Loads of them, all the time. I've seen kids that needed inhalers every two minutes and because they're doing this run, they're saying, I feel brilliant, I feel really good. And I've had that from, oh quite a few kids.* F1

***Concentration***

There were mixed, but slightly positive findings regarding how the Daily Mile affected pupils' concentration levels. Some teachers reported that they had not noticed any obvious changes in concentration levels since the Daily Mile was introduced.

For peer review only

*I definitely couldn't say that, you know, their concentration is better because I do it at weird times of the day. It doesn't help unfortunately. And I would love to do it halfway through the afternoon, but it's just not feasible. And to get them settled and back down again, I don't know if that's worse...* F2

However, others stated that they have observed noticeable improvements in concentration and would often choose to do the activity at specific time of the day when concentration levels are expected to be low.

*Doing it in that halfway between break time and lunchtime, you could see a dip in concentration coming, we would go outside, just ten minutes outside, we have a run around, we come back in and then we'd go back to our job and we were maybe a bit more prepared for a bit more learning and thinking.* B1

***Behaviour***

The impact of the Daily Mile on behaviour was a contentious issue amongst the participants. Some teachers argued that the Daily Mile made little or no difference to disruptive behaviour of pupils.

*I really didn't have behaviour issues last year, this year I've got loads of them and I can't honestly say that there is huge difference yet.* D1

*I still have the same disruptive behaviours on the days that I do it [the Daily Mile] as on the days that I don't do it, unfortunately.* E1

For peer review only

However, two teachers felt that the Daily Mile did improve behaviour in the classroom. They attributed this to the release of excess energy, which often made the children restless at certain times of the day. The example below by one of the teachers explains this point.

*And from a behavioural point of view there was one particular boy who was really switched off, you know, but the running really…he performed really well in that and it, sort of, really made a positive impact on how he felt about himself and how he performed generally in school.* C1

***Teacher-pupil rapport***

Approximately half of the participants stated that a major benefit of the Daily Mile was that it allowed them the opportunity to build a rapport with the children in their class in a way that is difficult to do in a classroom environment.

> *It's the pastoral side of it. Kids will tell you things while you're out there doing that that they won't tell you in a classroom setting, or that they won't disclose to another adult. Not, kind of, generally speaking, you know, kind of big scary disclosures, but they'll just be more open and honest with you.* A1

> *It's also nice for teachers, it gives you a chance to run alongside and walk alongside some of the children and have a chat about something that may have happened in class, or you just want to have a chat with them, because it's not something we get to do one to one in the class, we don't have time.* E2

Teachers acknowledged that the Daily Mile offered a unique opportunity to talk about issues with certain individuals, or to get to know the children in their class better.



For peer review only - http://bmjopen.bmj.com/site/about/guidelines.xhtml

*It's a really social time because you get a chance to speak to the kids, or you can deal with any issues. I think actually along with them running, because every lap, a couple of them might stop and chat for a second.* F2

***Perceived impact on learning time***

In general, the Daily Mile was considered a worthwhile initiative due to the known benefits of physical activity on health and wellbeing. However, the majority of the participants conceded that it is time-consuming, and reduces the amount of time they have available to dedicate to other subjects in the classroom per week, which may potentially affect learning.

*The biggest challenge is the fact that it takes out from other learning time, and that can be really challenging because most teachers are already feeling like they're trying to cram too much into the day. So to add another thing into the day is really difficult, even though you know it's important, you want them to do maths and read and write and all those other important skills as well. It's just trying to fit everything in.* E1

Furthermore, some participants were of the opinion that as the Daily Mile is not supposed to count as PE, they struggle to provide both activities in full to their class. Teachers stated that this was a contentious issue amongst colleagues when discussing the Daily Mile, and a number of participants mentioned that some colleagues refuse to do the Daily Mile or have complained about it due to time constraints in terms of delivering a full curriculum.

*We're so stressed for time. You have to do it every day. If you've got a PE lesson, then you have to have a Daily Mile as well.* D1

A number of teachers expressed the view that getting the children ready to go outside was time-consuming, and added additional time constraints in addition to the Daily Mile itself.

For peer review only

*The other issue is, these children here change their shoes and it takes so long to change shoes to go outside and change back that you're losing a chunk of time.* G1

***Weather and resources***

All the participants identified the weather as a barrier to participation; specifically wet and icy weather. However, the majority of teachers felt they would like to do the Daily Mile in all weathers, but are limited by unsafe running surfaces, and inappropriate footwear/weather gear worn by some children.

*We often have howling gales here and so we probably wouldn't go out in howling rain.* G1

As previously mentioned, some teachers highlighted the running surface as a limitation. Specifically, Schools with large greenspace were viewed as suitable for participation. However, this is not necessarily safe to run on in wet conditions, which limited the ability of some schools to do the Daily Mile in wet weather due to not having access to alternative facilities.

*There is a hard tarmac and there is a grass field, I mean, they use the grass field part of the playground as long as it's dry. As soon as it's wet we can't use that, so it's actually on a rough tarmac it's not a good running surface at all.* D2

Alternatively, teachers at the school with insufficient greenspace felt that this limited their ability to do the Daily Mile due to insufficient space and inadequate surfaces.

Teachers that had access to artificial playing surfaces or all-weather surface generally believed that their school had better resources for Daily Mile participation than those without.

For peer review only

*We can use it (3G pitch) in all weathers and, you know, they don't need to get changed, they are not having to worry about mud and things like that.* C1

While facilities were deemed important to participation, in general the teachers found ways to adapt the initiative to suit the facilities that were available at their school. This sometimes meant not participating in severe weather, or using indoor space such as a gym hall as an alternative.

Another barrier identified was that a number of teachers felt that some children were not properly equipped with the appropriate clothing to take part in the Daily Mile in all weathers.

*The kids don't all have wet weather gear. Loads of kids who don't have…like, I mean, this is the first week where all the children in my class have worn a coat. So I would say that's much more of a barrier than having the space.* E2

**Discussion**

In this qualitative study that examined teachers' perspectives of the barriers, facilitators and the perceived benefits of the Daily Mile, we found that teachers were generally positive about the initiative, and felt the intervention was warranted considering the health benefits of physical activity, and its potential to reduce childhood obesity. However, a considerable number of teachers were concerned about the time-consuming nature of the Daily Mile and the impact of this on learning time.

From 1998, the prevalence of overweight, including obesity amongst children aged 2-15 has fluctuated between 28% and 33%, and was 29% in 2016 [14].This high prevalence of overweight and obesity highlights the fact that existing interventions are not having the anticipated impact, at


For peer review only - http://bmjopen.bmj.com/site/about/guidelines.xhtml

least at the population level. Therefore, innovative school-based physical activity programmes such as the Daily Mile require attention in order to understand how they can contribute to the reduction of childhood obesity; which is a major target within current UK and international health policy [15]. Such programmes replace sedentary behaviour and light physical activity with MVPA and tend to reduce adiposity [16]. It is in this respect that the Scottish Government is promoting the Daily Mile initiative more widely across schools in Scotland. The concept of the Daily Mile has also generated international interest, yet until recently there was little evidence regarding its efficacy. The Chesham et al. (2018) study has provided some assurance of the Daily Mile's benefits to children in terms of increasing levels of MVPA, reducing sedentary time, increasing physical fitness and improving body composition. Certainly, it would be interesting to see whether such benefits can be sustained in the longer-term and how they translate into meaningful reductions in childhood obesity at the population level.

Although the Daily Mile follows a generic protocol and procedure for implementation, schools and teachers have autonomy over when and how they execute it. Our findings show that some teachers did not undertake the Daily Mile every school day. A growing evidence base is demonstrating that a dose-response relationship exists between physical activity and its associated health benefits. Specifically, improvements in health outcomes tend to increase along with the levels of physical activity [17-19]. Interestingly, some of the teachers in our study stated that they or their colleagues would occasionally count the Daily Mile as part of the compulsory two hours of quality physical education time, which may offset the benefits of having the Daily Mile as a stand-alone activity-as it is intended [8]. Despite this, Chesham et al. (2018) found that compared with control schools, the Daily Mile intervention schools increased MVPA by about 9 minutes per day, indicating that the intervention does increase PA and in turn may have potential



For peer review only - http://bmjopen.bmj.com/site/about/guidelines.xhtml

health benefits. Further research may be required to understand the nature and level of benefits that could be garnered for children within classes that do not undertake the Daily Mile on a more regular basis.

Despite the fact that teachers felt motivated to do the Daily Mile, the study also showed the Daily Mile increased pressure on teachers' workload and they were concerned about its detrimental effect on learning time. This was in contrast to a similar study in Italy, which also examined teachers and students' attitudes to an outdoor active break programme in a middle school [20]. The study reported that teachers found the outdoor active break programme easy to organise and that it did not negatively influence their teaching activities. It is possible that any difference in the attitudes of teachers in the aforementioned study and our study are due to the age of children involved. Whilst we used primary school teachers, with pupils usually aged 4.5 -11 years, the Italian study used middle school teachers, who taught students aged about 12 years. A recent historical account indicates that increasing focus on academic achievement decreases physical activity opportunities in schools [21]. With academic achievement under constant scrutiny in most countries, it appears that evidence to ascertain whether the Daily Mile promotes cognition and academic achievement could assure primary school teachers that time invested in the Daily Mile would engender additional educational benefits. Current evidence suggest that physical activity shows a positive effect on constructs related to academic achievement [21-23]. Perhaps contextualising this evidence in relation to performing the Daily Mile in addition to usual physical education curricular might be more relevant for primary school teachers.

Another important finding of the study was that Daily Mile offered social time for teachers and children. This was viewed as important in building a more supportive teacher-pupil relationship. A study has suggested that such supportive relationships are characterised by warmth, trust, and

For peer review only

low degrees of conflict and were associated with positive school outcomes for children with behavioural issues [24]. Positive teacher-pupil relationships can therefore improve student motivation to learn as well as their academic achievement [25]. The effect of the Daily Mile on teacher-pupil relationships is an interesting finding and should be explored further in future studies.

It was clear that the weather had implications in terms of participation for schools without adequate outdoor facilities. The Daily Mile is an outdoor school-based physical activity programme. Schools in temperate regions where the weather is often unpredictable during the winter months should consider improvement to their outdoor facilities in order to have the best chance of fully delivering the Daily Mile throughout the school year.

***Strengths and limitations***

The study used qualitative interviews to generate a deeper insight into the perspectives of teachers who have crucial role in the delivery of Daily Mile in Schools. However, only the accounts of teachers were used to understand these barriers and facilitators. Using additional data from pupils would have allowed triangulation of data, which would have enhanced our findings. We struggled to recruit teachers into the study, so it is possible that teachers who were likely in favour of the Daily Mile participated. This may imply that the level of positivity depicted by the study may not reflect the overall opinion of teachers who deliver the Daily Mile. On the contrary, it could also signal that any barriers identified were important concerns, which require attention as the Daily Mile continues to expand internationally. Future studies should measure the attitudes and practices of teachers to the Daily Mile using quantitative approaches such as surveys, which can reach a larger sample.


For peer review only - http://bmjopen.bmj.com/site/about/guidelines.xhtml

## Conclusion

Given that existing obesity prevention strategies are struggling to stem the tide of childhood obesity, innovative school-based physical activity interventions such as the Daily Mile present promising opportunities for policy change within education. However, any policy change should be underpinned by research if the practice of the Daily Mile is to be adopted more widely. This study has identified important barriers and facilitators from the perspectives of teachers who play a crucial role in the delivery of the Daily Mile. Our findings indicate that teachers were motivated to deliver the Daily Mile and were positive about its potential to improve physical activity and related health outcomes. However, they were also concerned that it was negatively affecting learning time.

## Funding details

This work was supported by a University of Edinburgh Innovation Initiative Grant under Grant number GR002292. The authors were both employed by the Scottish Collaboration for Public Health Research and Policy (SCPHRP), University of Edinburgh. At the time of the study, SCPHRP's core Grant was from the Medical Research Council (Grant Number MR/K023209/1) and the Chief Scientist Office of Scotland.

## Acknowledgements

We thank Marion Barclay for her assistance with recruitment for this study, and both Dr Josie Booth and Dr Gemma Ryde for their comments on the interview topic guide. We also thank all the participants who took part in our study.

## Disclosure statement

The authors declare that they have no competing interests.

## Contributorship statement

For peer review only

MB (physical activity coordinator) assisted SM and LD with recruitment for the study. SM and LD designed the study and conducted data collection with participants. Both authors analysed the data, and SM drafted the first draft of the manuscript. SM and LD prepared the final draft of the manuscript for submission.

**Data availability statement**

Ethical restriction by the University of Edinburgh Centre for Population Health Sciences Research Ethics Committee prohibits the authors from making the minimal data set publicly available because data contain potentially identifiable information. However, data are available from the corresponding author on reasonable request.

For peer review only

**References**

1. Janssen I, LeBlanc AG. Systematic review of the health benefits of physical activity and fitness in school-aged children and youth. *International journal of behavioral nutrition and physical activity* 2010;7(1):40.
2. Reilly JJ, Armstrong J, Dorosty AR, et al. Early life risk factors for obesity in childhood: cohort study. *Bmj* 2005;330(7504):1357.
3. Gurnani M, Birken C, Hamilton J. Childhood obesity: causes, consequences, and management. *Pediatric Clinics* 2015;62(4):821-40.
4. van Jaarsveld CH, Gulliford MC. Childhood obesity trends from primary care electronic health records in England between 1994 and 2013: population-based cohort study. *Archives of disease in childhood* 2015:archdischild-2014-307151.
5. Reilly JJ, Johnstone A, McNeill G, et al. Results from Scotland's 2016 Report card on physical activity for children and youth. *Journal of physical activity and health* 2016;13(11 Suppl 2):S251-S55.
6. Cooper AR, Goodman A, Page AS, et al. Objectively measured physical activity and sedentary time in youth: the International children's accelerometry database


For peer review only - http://bmjopen.bmj.com/site/about/guidelines.xhtml

(ICAD). *International journal of behavioral nutrition and physical activity* 2015;12(1):113.

7. Scottish Executive. A curriculum for excellence: Edinburgh: Scottish Executive, 2004.

8. The Daily Mile Foundation. About The Daily Mle Lyndhurst: The Daily Mile Foundation; 2018 [Available from: https://thedailymile.co.uk/about/ accessed 03/08/2018 2018.

9. Chesham RA, Booth JN, Sweeney EL, et al. The Daily Mile makes primary school children more active, less sedentary and improves their fitness and body composition: a quasi-experimental pilot study. *BMC medicine* 2018;16(1):64.

10. Breheny K, Adab P, Passmore S, et al. A cluster randomised controlled trial evaluating the effectiveness and cost-effectiveness of the daily mile on childhood obesity and wellbeing; the Birmingham daily mile protocol. *BMC public health* 2018;18(1):126.

11. Pawson R, Tilley N. An introduction to scientific realist evaluation. 1997

12. Richmond SA, Donaldson A, Macpherson A, et al. Facilitators and Barriers to the Implementation of iSPRINT: A Sport Injury Prevention Program in Junior High Schools. *Clinical journal of sport medicine: official journal of the Canadian Academy of Sport Medicine* 2018

13. Executive S. Scottish Index of Multiple Deprivation: Scottish Executive Edinburgh, 2006.

14. Scottish Government. Health of Scotland's Population- Obesity Edinburgh: The Scotttish Government; 2017 [Available from: https://www.gov.scot/Topics/Statistics/Browse/Health/TrendObesity accessed 21/05/2018 2018.

15. Scottish Government. A Healthier Future –Scotland’s Diet & Healthy Weight Delivery Plan. Edinburgh, 2018.

16. Fairclough SJ, Dumuid D, Mackintosh KA, et al. Adiposity, fitness, health-related quality of life and the reallocation of time between children's school day activity behaviours: A compositional data analysis. *Preventive medicine reports* 2018;11:254-61.

17. Foulds HJ, Bredin SS, Charlesworth SA, et al. Exercise volume and intensity: a dose–response relationship with health benefits. *European journal of applied physiology* 2014;114(8):1563-71.

18. Loprinzi PD, Lee H, Cardinal BJ. Dose response association between physical activity and biological, demographic, and perceptions of health variables. *Obesity facts* 2013;6(4):380-92.

19. Williams PT. Dose-response relationship of physical activity to premature and total all-cause and cardiovascular disease mortality in walkers. *PLoS One* 2013;8(11):e78777.

20. Brustio PR, Moisè P, Marasso D, et al. Feasibility of implementing an outdoor walking break in Italian middle schools. *PloS one* 2018;13(8):e0202091.

21. Howie EK, Pate RR. Physical activity and academic achievement in children: A historical perspective. *Journal of sport and health science* 2012;1(3):160-69.

22. McPherson A, Mackay L, Kunkel J, et al. Physical activity, cognition and academic performance: an analysis of mediating and confounding relationships in primary school children. *BMC public health* 2018;18(1):936.

For peer review only

23. Rasberry CN, Lee SM, Robin L, et al. The association between school-based physical activity, including physical education, and academic performance: a systematic review of the literature. *Preventive medicine* 2011;52:S10-S20.
24. Baker JA, Grant S, Morlock L. The teacher-student relationship as a developmental context for children with internalizing or externalizing behavior problems. *School Psychology Quarterly* 2008;23(1):3.
25. Yunus MM, Osman WSW, Ishak NM. Teacher-student relationship factor affecting motivation and academic achievement in ESL classroom. *Procedia-Social and Behavioral Sciences* 2011;15:2637-41.

For peer review only

For peer review only



For peer review only - http://bmjopen.bmj.com/site/about/guidelines.xhtml

Appendix 1. Daily Mile Interview Topic Guide

Does the Daily Mile take place at this school? If so, can you give us a brief history of its use at the school? (Were you given any assistance/guidance when setting it up?)

Can you describe a typical day at school for your class- when does the Daily Mile come into play?

What is your understanding of what the Daily Mile actually is? (what does it involve, what are its objectives etc)

When it was first introduced, how did you feel about it? (and have your feelings changed since its implementation?)

Is physical activity a major focus at this school? *(Probe for any physical activity policies that are in place, what does physical education involve/how often is it, is the playground appropriate for physical activity).*

Do you think the children that you teach should be more physically active? *(Are there noticeable issues related to physical activity in the class, obesity, generally unfit etc.).*

In general, how well are the children you teach performing academically? *( are there many children with additional support needs?*

Are there any behavioural issues in the class? *(How well do they listen and follow instructions? Are there any particular times of the day when they are more disruptive/easier or harder to teach?)*

Does their behaviour change following the Daily Mile at all?

Are there any wider issues within the community in which this school is located that you think may hinder/enhance the achievement of children at school? *(Look to discuss the deprivation/affluence of the area, level of engagement with children's learning and development from parents, area safety and crime, provision of adequate facilities. Do these same issues, or others, possibly impact on the children's physical activity levels?).*

Considering all the things we have just discussed, what effect do you think the Daily Mile programme would have on children at this school? *(How would it effect their physical activity levels, would this benefit their overall health? Do you think it would have any effects on their learning or behaviour in class?).*

Thinking practically about what the Daily Mile programme would actually involve, how feasible was it to implement the Daily Mile programme at this school, and keep it running on a daily basis? *(Do you think it is worth sacrificing the classroom time to do it? Do you think other teachers at this school would be in favour of doing the Daily Mile with their class?).*

Were you aware of any challenges that came to light as the Daily Mile was being implemented?

What have been the main barriers for implementing this programme here?

The first school to use the Daily Mile has an all-weather track around the playground for children to use. Do you think the playground at this school is adequate for the Daily Mile to

For peer review only

take place? *(Is there enough space? Would you need to build an all-weather track? What other resources do you think would be required to implement and sustain the programme here?)*

What specific benefits do you think the Daily Mile is having on children at this school? *(Health benefits, academic benefits, behavioural benefits?)*

How do you think parents feel about their children taking part in the Daily Mile, are they aware of it?

What do you think the children think of the Daily Mile?

Considering everything we have discussed, and the specific issues that are present in this area and at this school do you think the introduction of the Daily Mile has been a positive thing overall?

What has been the response from your colleagues?

For peer review only

BMJ Open

# The Daily Mile: Teachers' perspectives of the barriers and facilitators to the delivery of a school-based physical activity intervention

| | |
|---|---|
| Journal: | *BMJ Open* |
| Manuscript ID | bmjopen-2018-027169.R1 |
| Article Type: | Research |
| Date Submitted by the Author: | 22-Dec-2018 |
| Complete List of Authors: | Malden, Stephen; University of Edinburgh, Scottish Collaboration for Public Health Research and Policy, School of Health in Social Science, Doorway 6, Old Medical School, Teviot Place, University of Edinburgh, Edinburgh ; University of Strathclyde School of Psychological Sciences and Health, Physical Activity for Health Group<br>Doi, Lawrence; University of Edinburgh, Scottish Collaboration for Public Health Research and Policy, School of Health in Social Science, Doorway 6, Old Medical School, Teviot Place, University of Edinburgh |
| <b>Primary Subject Heading</b>: | Public health |
| Secondary Subject Heading: | Paediatrics |
| Keywords: | physical activity, daily mile, education, teacher, school |
| | |

SCHOLARONE™
Manuscripts

# The Daily Mile: Teachers' perspectives of the barriers and facilitators to the delivery of a school-based physical activity intervention

Stephen Malden[1,2] stephen.malden@strath.ac.uk,

Lawrence Doi[1] larry.doi@ed.ac.uk

[1]Scottish Collaboration for Public Health Research and Policy, School of Health in Social Science, Doorway 6, Old Medical School, Teviot Place, University of Edinburgh, Edinburgh EH8 9AG, UK

[2]Physical Activity for Health Group, School of Psychological Sciences and Health, Graham Hills Building, room 535, 40 George Street, University of Strathclyde, Glasgow G1 1QE, UK

Corresponding author: Stephen Malden

Word count: 5833 words

For peer review only

**Abstract**

**Objectives:** Children spend a significant amount of their time in a school environment, often engaged in sedentary activities. The Daily Mile is a physical activity intervention, which aims to increase physical activity and fitness in children through the completion of an outdoor teacher-led walk or run during the school day. This study aimed to explore the barriers, facilitators and perceived benefits of the Daily Mile from the perspectives of teachers through the use of qualitative semi-structured interviews. It also aimed to identify important context-specific factors, which might require consideration for those who intend to adopt the Daily Mile.

**Setting:** Eight Local Authority primary schools in the City of Edinburgh and East Lothian, UK.

**Participants:** Thirteen teachers (eleven women) who teach children in primaries one to seven in a school which delivered the Daily Mile.

**Results:** Data were analysed using an interpretative thematic analysis. Teachers were positive and enthusiastic about the Daily Mile and perceived it to be beneficial to children's health and fitness. A number of barriers to participation were identified including inadequate all-weather running surfaces and time constraints in an already full school curriculum. The perceived impact on learning time was identified as a concern for teachers, while other benefits were also identified including increased teacher-child rapport and perceived enhanced classroom concentration levels.

**Conclusion:** The Daily Mile appears to be a valuable addition to the school day, however important context-specific barriers to delivery of the Daily Mile exist, which should be considered when implementing the Daily Mile in schools.

**Keywords:** Physical activity, daily mile, education, teacher, school

For peer review only

**Strengths and limitations of the study**

- One of the first studies to investigate teachers' views of the Daily Mile
- Sampled teachers from schools in multiple locations to investigate contextual factors
- Data collection with children wold have allowed for data triangulation
- Small sample size may limit generalisability of findings

**Introduction**

A lack of physical activity is a determinant of numerous health conditions in childhood [1]. Specifically, sedentary behaviours and low levels of physical activity are partly to blame for the marked increases in childhood obesity that have been observed internationally in recent years [2 3]. In the United Kingdom, childhood overweight and obesity levels are high, approximately 30% [4]. Additionally, physical activity levels are low [5] and have been shown to decrease with age, a trend that has been similarly observed in other European countries and the United States [6]. Considering this, there is a need for the implementation of effective, evidence-based interventions to increase physical activity levels in school-aged children.

The Daily Mile is an example of a school-based intervention that involves teachers taking their class outside at some point during the school day to participate in 15 minutes of exercise. Children choose what pace they complete the activity, with those who run for the full 15 minutes likely to have completed approximately one mile (approximately 1.6 kilometres). The intervention is intended to be delivered in addition to the 2 hours per week of quality physical education as required by the Scottish Curriculum [7], and is not intended to replace this time. The Daily Mile was originally introduced to primary schools (which teach ages ~5 to ~11 years) to increase fitness levels [8], but it may also have a positive effect on physical activity, sedentary behaviour and body composition [9]. Since its inception at St Ninian's Primary School in Stirling in 2012, participation and expansion of the initiative has grown exponentially, with the Daily Mile now taking place in more primary schools across Scotland, in addition to a number of sites both within the UK and internationally [8].



For peer review only - http://bmjopen.bmj.com/site/about/guidelines.xhtml

A recent quasi-experimental pilot study by Chesham et al. (2018) showed that when compared to control schools, children who participated in the Daily Mile showed significant improvements in objectively-measured daily moderate-vigorous physical activity (MVPA), objectively-measured sedentary time, fitness and body composition [9]. To date, the aforementioned study is the only research currently offering empirical evidence of the Daily Mile's efficacy, although another randomised trial is being planned [10]. However, interventions do not usually function the same everywhere, and it is important to understand contextual factors that may influence how they work [11]. Contextual issues have been found useful in other school programmes, where context-specific differences have been considered when testing and developing an intervention [12]. Furthermore, a systematic review of studies which examined factors which influenced the implementation of physical activity policies in schools, found that factors relating to environmental context/resources such as provision of adequate equipment/facilities, and social influences such as teacher/staff support were important predictors of implementation success or failure[13]. Therefore, examining the experiences and perspectives of teachers in early adopter schools would allow policy makers and practitioners to adapt the Daily Mile to suit their settings where necessary and identify facilitators and barriers to implementation.

A recent qualitative study by Ryde et al. (2018) was the first to assess the context specific factors, which influence the success of the Daily Mile's implementation in four schools in central Scotland. The study concluded that having a supportive organisational environment and allowing teachers autonomy to deliver the initiative facilitated the implementation of the intervention. While the study offers important insight into factors influencing the implementation of the Daily Mile, the authors highlight that it may be biased due to the included teachers being advocates of the Daily Mile and the selection of schools in one geographical area[14]. The authors recommended for future studies to interview more teachers



For peer review only - http://bmjopen.bmj.com/site/about/guidelines.xhtml

across different geographical locations in schools which had implemented the Daily Mile with varying degrees of success[14]. Therefore, the present study explored the barriers, facilitators and perceived benefits of the Daily Mile from the perspectives of teachers who deliver the intervention in multiple schools across two geographical locations in Scotland. It also aimed to identify important context-specific factors, which might require consideration for those who intend to adopt the Daily Mile in the future.

## Methods

### *Patient and Public Involvement*

A senior member of teaching staff (physical education coordinator) who had well established contacts with teachers in multiple schools acted as a liaison during recruitment and liaised with potential participants. The research findings will be shared with the participants in a short summary report which will be emailed to them directly and to head-teachers of the participating schools.

### *Design*

Adopting interpretive approach, qualitative methods were used to explore teachers' perspectives of the Daily Mile and their experiences of delivering it. Semi-structured interviews were conducted, consisting of open-ended questions which aimed to determine how teachers define the Daily Mile, how they deliver the initiative, perceived benefits for the children who participate, and potential context-specific barriers and facilitators to participation. Our goal was to explore their experiences of the Daily Mile in order to inform future practice rather than to develop new theory.

For peer review only

Ethical approval was granted by The University of Edinburgh's Usher Research Ethics Group, and Local Authority research in schools permission was obtained prior to recruitment. All participants were given an information sheet at the start of the study, before providing written informed consent.

***Setting, participants and recruitment***

This study was conducted in Local Authority primary schools within two council areas: the City of Edinburgh and East Lothian, Scotland. The sites contain significant rural and urban populations, while also having both some of the most affluent areas in the country and some of the most deprived areas, as defined by the Scottish Index of Multiple Deprivation (SIMD) in Table 1 [15].

Participants were primary school teachers who taught years 1 through to 7 at schools which either currently, or formerly, participated in the Daily Mile. As it is mostly class teachers who deliver the Daily Mile, head-teachers, or teaching staff who do not have regular prolonged contact with children were excluded from participating. A total of thirteen teachers took part in the study, consisting of eleven women and two men.

A purposive sampling strategy was used to recruit teachers. Specifically, contact was made with a physical education coordinator in one of the Local Authority areas who acted as a liaison between the research team and schools/teachers. Between August 2016 and February 2017, the liaison delivered study information sheets and personally spoke to selected head-teachers about the study. Schools/head teachers were approached based on the liaison's knowledge that they were a school that had been delivering the Daily Mile.  In order to enhance participants' recruitment, emails and letters were sent to head teachers of additional primary schools. This contained study information sheets and researcher's contact details. Twenty-one schools were approached to participate in the study, for which ten initially

For peer review only

agreed to participate. Teachers from eight of the ten schools subsequently agreed to participate in the study.

### *Data collection*

Semi-structured interviews were conducted face-to-face in a private room within the participating schools by one researcher. Both researchers have extensive experience in conducting qualitative interviews with a broad range of participants for public health evaluation purposes, and have undertaken formal training in qualitative data collection. Participants were asked to give a brief history of the Daily Mile at their school, before probing questions were asked regarding subjects such as how the initiative is viewed by the staff at the school, approaches taken to deliver the program, and any perceived benefits, facilitators, or barriers associated with participation and delivery. A sample of the interview topic guide can be viewed in appendix 1. Interviews were usually conducted after the school day and lasted between 21 and 58 minutes. All interviews were audio recorded via digital audio recorder and transcribed verbatim.

### *Data analysis*

Utilising interpretive approach, this study employed thematic analysis[16] to analyse the qualitative data that were generated following verbatim data transcription. After familiarising ourselves with the transcribed data, a coding framework was developed by both authors who independently coded the same two most informative transcripts from the dataset, before collaborating to refine the coding framework. Both authors independently read and re-read



For peer review only - http://bmjopen.bmj.com/site/about/guidelines.xhtml

each remaining transcript and used the coding framework to assign codes to the text. New codes identified were discussed and added to the framework. This ensured consistency and rigour. Subsequently, similar codes were grouped together into overarching themes, which were reviewed and refined to ensure the coded data adequately reflected themes that encompassed them. Interpretation of data was undertaken by comparing similarities and differences within and across themes. Data analysis was facilitated by NVivo 10 qualitative data analysis software.

## Results

### Characteristics of schools

The characteristics of the eight schools where the thirteen teachers were drawn from varied with regards to facilities, rurality and level of deprivation as detailed in Table 1. All but one school had access to sufficient greenspace within the school grounds, while half of the schools had access to an all-weather surface such as an Astroturf football pitch or a solid polyurethane running track. Three schools were located within a large town or city, three were in a semi-rural location such as a small country town, and two schools were in a rural location. Three schools were located within areas of high deprivation, three within areas of medium deprivation and two in affluent areas.

For peer review only

Table 1. Characteristics of schools

| School Identifier | Accessible greenspace in school grounds | Accessible all-weather surface in school grounds (such as Astroturf) | Rurality (rural, semi-rural, urban) | Level of deprivation (SIMD quintile, 1= most deprived 5= least deprived) |
|---|---|---|---|---|
| A | Yes | No | Semi-rural | 3 |
| B | Yes | Yes | Semi-rural | 5 |
| C | Yes | Yes | Urban | 3 |
| D | Yes | No | Rural | 4 |
| E | Yes | No | Urban | 1 |
| F | No | Yes | Urban | 1 |
| G | Yes | No | Rural | 5 |
| H | Yes | Yes | Semi-rural | 1 |

**Themes**

Eight themes were identified, and are detailed below.

***Style of delivery***

The style of delivery varied considerably between teachers and schools. While each school followed a similar Daily Mile protocol for delivery, some schools offered more autonomy to teachers concerning how and when they delivered the Daily Mile.

> *So yeah, it does happen in this school and what we do is that we split it, we don't do the mile in one go, we do half a mile in the morning and half a mile in the afternoon*

For peer review only

*and it's immediately after break, or immediately after lunch, with the exception of Thursdays when it really works in our favour because we do it immediately after assembly. Because the kids have been sitting down and just not, being in a stuffy hall for, you know, half an hour.* F2

There were also some differences in the frequency and level of engagement with the Daily Mile amongst the schools. A number of participants stated that all classes at their school took part, often at the request of the head teacher, while others mentioned that only some classes did the Daily Mile, and that participation does not happen every day either due to teacher preferences or age/ability of the children.

*Some of the other classes do it every day as well, but some just do it every now and then. F1*

*I know that the [primary] ones certainly don't do it because it's a lot more difficult to manage.* B2

There were slight variations in how the Daily Mile was defined by the teachers. Variations in what constitutes participation were evident, with some teachers defining the Daily Mile as 15 minutes of exercise, and others as an exact mile that is done to completion regardless of time taken. The emphasis on running the Daily Mile also differed between teachers and schools, with some teachers' strongly encouraging running, while others were happy for children to choose whether to walk or run.

*I don't start by saying, you can run or walk it, I start by saying, it's a run and I sneakily tell people they can walk if I think that, you know, they need to. But even if you tell them to walk, quite often they end up running.* D2

The time at which the Daily Mile was delivered during the school day also differed amongst the teachers. Some participants stated that they purposefully delivered the intervention at set



For peer review only - http://bmjopen.bmj.com/site/about/guidelines.xhtml

times during the day in order to establish a routine or get a desired behavioural or performance-based response from the children.

> *I'm doing it after break and lunch. I think that's really effective because you don't need to take them out. There's no difference. They're already outside. They can take their jackets off or they can leave them on.* F1

> *I've done it in the middle of the afternoon rather than straight after break – I much prefer that. I prefer to use it as a break in the lessons or change of lesson.* D1

There was variability amongst the sample regarding the interaction between the Daily Mile, and formal physical education sessions. A number of teachers explicitly stated that they do the Daily Mile in addition to the 2 hours per week of physical education recommended by the school curriculum. However, a few of the teachers asserted that they count the Daily Mile as part of the physical education curriculum and therefore as a part of the 2 hours of weekly PE which they are expected to deliver.

> *See, I didn't know that [the Daily Mile does not count towards PE time]. I've sometimes done it as a warm-up, so I've done the Daily Mile and then 45 minutes of PE and then don't have to do a warm up in PE.* D1

> *I think people were concerned about where would it fit, in the timetable, because obviously we're stretched for our two hours of PE, and now we have to do the Daily Mile. Last year I did it as part of my PE.* B2

***Motivating children to participate***

Approximately half of the teachers used some form of motivational or incentive/rewards-based system with children when doing the Daily Mile. Such techniques included the handing out of tokens for each lap of the running area completed, or tracking the distance ran over an



For peer review only - http://bmjopen.bmj.com/site/about/guidelines.xhtml

accumulative time-period and matching it with the distances to various geographical locations.

> *With the Primary 1s and 2s and 3s last year, we gave them…you know the Unifix Cubes that stick together? So every time they complete a lap, they get a cube. And they add them on. And it was good in so far as we could use that then as a numeracy lesson and obviously for the little ones, you know, they were counting, they were being inspired.* D1

> *We have a big board at the front of the school and it shows everybody's progress tracked. We are aiming to go to different places in the world and to the moon as a whole school. It's really positive and they are motivated.* H1

Some teachers stated that they found that their own participation in the Daily Mile motivated the children to try to keep up or beat them, thus increasing the children's engagement with the activity.

> *Every now and again I'll go and run with them. But they'll always, you always see a boost in them when, "the teacher's coming, the teacher's coming, he's coming!" And then I'll just go, oh come on guys, I'm beating you and I'm so much older. And it just adds something to it.* F2

***Health, wellbeing and fitness***

A number for the teachers attributed the Daily Mile to improvements in general health and fitness that they observe amongst children in their care. Specifically, the majority of the teachers indicated that the children's time taken to complete the Daily Mile had generally


For peer review only - http://bmjopen.bmj.com/site/about/guidelines.xhtml

improved since the programme started, and others attributed school success in sporting events to Daily Mile participation

*We interviewed some of the children about how they'd got on [competing in an athletics competition]. Actually their reflections were, we think we did better because we are fitter because we do the mile and that wasn't prompted at all, so I think that they feel fitter and healthier.* C1

*They are definitely fitter because I forgot to say that we are recording last year's Primary 5s at the start of the year and we did fitness levels with them before we even started the mile. We've done the same cohort of children every term since and we have that recorded as well for every single child's results we can see that they are all fitter, so that's pretty good.* H1

Two teachers also speculated that Daily Mile participation led to improvements in asthma symptoms amongst some children. One of the teachers explained this below.

*I'm seeing a great difference in kids with asthma. Huge. Loads of them, all the time. I've seen kids that needed inhalers every two minutes and because they're doing this run, they're saying, I feel brilliant, I feel really good. And I've had that from, oh quite a few kids.* F1

***Concentration***

There were mixed, but slightly positive findings regarding how the Daily Mile affected pupils' concentration levels. Some teachers reported that they had not noticed any obvious changes in concentration levels since the Daily Mile was introduced.



For peer review only - http://bmjopen.bmj.com/site/about/guidelines.xhtml

*I definitely couldn't say that, you know, their concentration is better because I do it at weird times of the day. It doesn't help unfortunately. And I would love to do it halfway through the afternoon, but it's just not feasible. And to get them settled and back down again, I don't know if that's worse...* F2

However, others stated that they have observed noticeable improvements in concentration and would often choose to do the activity at specific time of the day when concentration levels are expected to be low.

*Doing it in that halfway between break time and lunchtime, you could see a dip in concentration coming, we would go outside, just ten minutes outside, we have a run around, we come back in and then we'd go back to our job and we were maybe a bit more prepared for a bit more learning and thinking.* B1

***Behaviour***

The impact of the Daily Mile on behaviour was a contentious issue amongst the participants. Some teachers argued that the Daily Mile made little or no difference to disruptive behaviour of pupils.

*I really didn't have behaviour issues last year, this year I've got loads of them and I can't honestly say that there is huge difference yet.* D1

*I still have the same disruptive behaviours on the days that I do it [the Daily Mile] as on the days that I don't do it, unfortunately.* E1

However, two teachers felt that the Daily Mile did improve behaviour in the classroom. They attributed this to the release of excess energy, which often made the children restless at certain times of the day. The example below by one of the teachers explains this point.

For peer review only

*And from a behavioural point of view there was one particular boy who was really switched off, you know, but the running really…he performed really well in that and it, sort of, really made a positive impact on how he felt about himself and how he performed generally in school.* C1

***Teacher-pupil rapport***

Approximately half of the participants stated that a major benefit of the Daily Mile was that it allowed them the opportunity to build a rapport with the children in their class in a way that is difficult to do in a classroom environment.

> *It's the pastoral side of it. Kids will tell you things while you're out there doing that that they won't tell you in a classroom setting, or that they won't disclose to another adult. Not, kind of, generally speaking, you know, kind of big scary disclosures, but they'll just be more open and honest with you.* A1

> *It's also nice for teachers, it gives you a chance to run alongside and walk alongside some of the children and have a chat about something that may have happened in class, or you just want to have a chat with them, because it's not something we get to do one to one in the class, we don't have time.* E2

Teachers acknowledged that the Daily Mile offered a unique opportunity to talk about issues with certain individuals, or to get to know the children in their class better.

> *It's a really social time because you get a chance to speak to the kids, or you can deal with any issues. I think actually along with them running, because every lap, a couple of them might stop and chat for a second.* F2

***Perceived impact on learning time***

For peer review only

In general, the Daily Mile was considered a worthwhile initiative due to the known benefits of physical activity on health and wellbeing. However, the majority of the participants conceded that it is time-consuming, and reduces the amount of time they have available to dedicate to other subjects in the classroom per week, which may potentially affect learning.

> *The biggest challenge is the fact that it takes out from other learning time, and that can be really challenging because most teachers are already feeling like they're trying to cram too much into the day. So to add another thing into the day is really difficult, even though you know it's important, you want them to do maths and read and write and all those other important skills as well. It's just trying to fit everything in.* E1

Furthermore, some participants were of the opinion that as the Daily Mile is not supposed to count as PE, they struggle to provide both activities in full to their class. Teachers stated that this was a contentious issue amongst colleagues when discussing the Daily Mile, and a number of participants mentioned that some colleagues refuse to do the Daily Mile or have complained about it due to time constraints in terms of delivering a full curriculum.

> *We're so stressed for time. You have to do it every day. If you've got a PE lesson, then you have to have a Daily Mile as well.* D1

A number of teachers expressed the view that getting the children ready to go outside was time-consuming, and added additional time constraints in addition to the Daily Mile itself.

> *The other issue is, these children here change their shoes and it takes so long to change shoes to go outside and change back that you're losing a chunk of time.* G1

***Weather and resources***



For peer review only - http://bmjopen.bmj.com/site/about/guidelines.xhtml

All the participants identified the weather as a barrier to participation; specifically wet and icy weather. However, the majority of teachers felt they would like to do the Daily Mile in all weathers, but are limited by unsafe running surfaces, and inappropriate footwear/weather gear worn by some children.

> *We often have howling gales here and so we probably wouldn't go out in howling rain.* G1

As previously mentioned, some teachers highlighted the running surface as a limitation. Specifically, schools with large greenspace were viewed as suitable for participation. However, this is not necessarily safe to run on in wet conditions, which limited the ability of some schools to do the Daily Mile in wet weather due to not having access to alternative facilities.

> *There is a hard tarmac and there is a grass field, I mean, they use the grass field part of the playground as long as it's dry. As soon as it's wet we can't use that, so it's actually on a rough tarmac it's not a good running surface at all.* D2

Alternatively, teachers at the school with insufficient greenspace felt that this limited their ability to do the Daily Mile due to insufficient space and inadequate surfaces.

Teachers that had access to artificial playing surfaces or all-weather surface generally believed that their school had better resources for Daily Mile participation than those without.

> *We can use it (3G pitch) in all weathers and, you know, they don't need to get changed, they are not having to worry about mud and things like that.* C1

While facilities were deemed important to participation, in general the teachers found ways to adapt the initiative to suit the facilities that were available at their school. This sometimes



For peer review only - http://bmjopen.bmj.com/site/about/guidelines.xhtml

meant not participating in severe weather, or using indoor space such as a gym hall as an alternative.

Another barrier identified was that a number of teachers felt that some children were not properly equipped with the appropriate clothing to take part in the Daily Mile in all weathers.

> *The kids don't all have wet weather gear. Loads of kids who don't have…like, I mean, this is the first week where all the children in my class have worn a coat. So I would say that's much more of a barrier than having the space.* E2

**Discussion**

The Daily Mile offers an innovative approach to Physical Activity in schools, and similar to another recent study [14], this study has identified important factors that influence the implementation of the Daily Mile, and has added further to the knowledge-base regarding the context-specific barriers and facilitators related to the intervention from the perspectives of teachers. We found that teachers were generally positive about the initiative, and felt the intervention was warranted considering the health benefits of physical activity, and its potential to reduce childhood obesity. However, a considerable number of teachers were concerned about the time-consuming nature of the Daily Mile and the impact of this on learning time.

Although the Daily Mile follows a generic protocol and procedure for implementation, schools and teachers have autonomy over when and how they execute it. Our findings show that some teachers did not undertake the Daily Mile every school day. A growing evidence base is demonstrating that a dose-response relationship exists between physical activity and its associated health benefits. Specifically, improvements in health outcomes tend to increase along with the levels of physical activity [17-19]. Interestingly, some of the teachers in our study


For peer review only - http://bmjopen.bmj.com/site/about/guidelines.xhtml

stated that they or their colleagues would occasionally count the Daily Mile as part of the compulsory two hours of quality physical education time, which may offset the benefits of having the Daily Mile as a stand-alone activity-as it is intended [8]. Additionally, such practices may reduce the wider benefits associated with physical education such as motor skill development and the social/cognitive benefits of sport and team games which may not be addressed by Daily Mile participation[20]. Despite this, Chesham et al. (2018) found that compared with control schools, the Daily Mile intervention schools increased MVPA by about 9 minutes per day, indicating that the intervention does increase PA and in turn may have potential health benefits. However, whether similar findings would be observed in schools, which participate in the intervention less regularly, or combine the Daily Mile with physical education time may merit further investigation.

Despite the fact that teachers felt motivated to deliver the Daily Mile, the study also showed the Daily Mile increased pressure on teachers' workload and they were concerned about its detrimental effect on learning time; a finding that is supported by studies of other school-based physical activity initiatives[21 22]. This was in contrast to a similar study in Italy, which also examined teachers and students' attitudes to an outdoor active break programme in a middle school [23]. The study reported that teachers found the outdoor active break programme easy to organise and that it did not negatively influence their teaching activities. It is possible that any differences in the attitudes of teachers in the aforementioned study and our study are due to the age of children involved. Whilst we used primary school teachers, with pupils usually aged 5 -11 years, the Italian study used middle school teachers, who taught students aged about 12 years. A recent historical account indicates that increasing focus on academic achievement decreases physical activity opportunities in schools [24]. With academic achievement under constant scrutiny in most countries, it appears that evidence to ascertain whether the Daily Mile promotes cognition and academic achievement could assure primary



For peer review only - http://bmjopen.bmj.com/site/about/guidelines.xhtml

school teachers that time invested in the Daily Mile would engender additional educational benefits. Current evidence suggest that physical activity can show a positive effect on constructs related to academic achievement [24-26]. However, recent studies have also demonstrated that academic achievement is more strongly associated with sedentary time than physical activity [27 28]. These conflicting findings highlight the fact that at present, little is known regarding the optimum balance of sedentary time and physical activity for both academic performance and health. Future evaluations of school-based PA interventions similar to the Daily Mile should therefore incorporate measures of academic performance into their design.

Another important finding of the study was that Daily Mile offered social time for teachers and children. This was viewed as important in building a more supportive teacher-pupil relationships. A study has suggested that such supportive relationships are characterised by warmth, trust, and low degrees of conflict and were associated with positive school outcomes for children with behavioural issues [29]. Positive teacher-pupil relationships can therefore improve student motivation to learn as well as their academic achievement [30]. The effect of the Daily Mile on teacher-pupil relationships is an interesting finding and should be explored further in future studies.

It was clear that the weather had implications in terms of participation for schools without adequate outdoor facilities, which is consistent with the findings of the review by Nathan et al[13] which demonstrated that factors such as inadequate facilities and adverse weather were identified as barriers to implementation of school-based interventions in seven studies. The Daily Mile is an outdoor school-based physical activity programme. Therefore, schools in temperate regions where the weather is often unpredictable during the winter months should consider improvement to their outdoor facilities in order to have the best chance of fully delivering the Daily Mile throughout the school year.



For peer review only - http://bmjopen.bmj.com/site/about/guidelines.xhtml

From 1998, the prevalence of overweight, including obesity amongst children aged 2-15 has fluctuated between 28% and 33%, and was 29% in 2016 [31].This high prevalence of overweight and obesity highlights the fact that existing interventions are not having the anticipated impact, at least at the population level. Therefore, innovative school-based physical activity programmes such as the Daily Mile require attention in order to understand how they can contribute to the reduction of childhood obesity; which is a major target within current UK and international health policy [32]. Such programmes replace sedentary behaviour and light physical activity with MVPA and tend to reduce adiposity [33]. It is in this respect that the Scottish Government is promoting the Daily Mile initiative more widely across schools in Scotland. The concept of the Daily Mile has also generated international interest, yet until recently there was little evidence regarding its efficacy. The Chesham et al. (2018) study has provided some assurance of the Daily Mile's benefits to children in terms of increasing levels of MVPA, reducing sedentary time, increasing physical fitness and improving body composition. Certainly, it would be interesting to see whether such benefits can be sustained in the longer-term and how they translate into meaningful reductions in childhood obesity at the population level.

***Strengths and limitations***

The study used qualitative interviews to generate a deeper insight into the perspectives of teachers who have crucial role in the delivery of Daily Mile in Schools. However, only the accounts of teachers were used to understand these barriers and facilitators. Using additional data from pupils would have allowed triangulation of data, which would have enhanced our findings. We struggled to recruit teachers into the study, so it is possible that teachers who were likely in favour of the Daily Mile participated. This may imply that the level of positivity depicted by the study may not reflect the overall opinion of teachers who deliver the Daily Mile. On the contrary, it could also signal that any barriers identified were



For peer review only - http://bmjopen.bmj.com/site/about/guidelines.xhtml

For peer review only

important concerns, which require attention as the Daily Mile continues to expand internationally. Future studies should measure the attitudes and practices of teachers to the Daily Mile using quantitative approaches such as surveys, which can reach a larger sample.

**Conclusion**

Given that existing obesity prevention strategies are struggling to stem the tide of childhood obesity, innovative school-based physical activity interventions such as the Daily Mile present promising opportunities for policy change within education. However, any policy change should be underpinned by research if the practice of the Daily Mile is to be adopted more widely. This study has identified important barriers and facilitators from the perspectives of teachers who play a crucial role in the delivery of the Daily Mile. Our findings indicate that teachers were motivated to deliver the Daily Mile and were positive about its potential to improve physical activity and related health outcomes. However, they were also concerned that it was negatively affecting learning time.

**Funding details**

This work was supported by a University of Edinburgh Innovation Initiative Grant under Grant number GR002292. The authors were both employed by the Scottish Collaboration for Public Health Research and Policy (SCPHRP), University of Edinburgh. At the time of the study, SCPHRP's core Grant was from the Medical Research Council (Grant Number MR/K023209/1) and the Chief Scientist Office of Scotland.

**Acknowledgements**

We thank Marion Barclay for her assistance with recruitment for this study, and both Dr Josie Booth and Dr Gemma Ryde for their comments on the interview topic guide. We also thank all the participants who took part in our study.

**Disclosure statement**

The authors declare that they have no competing interests.



For peer review only - http://bmjopen.bmj.com/site/about/guidelines.xhtml

## Contributorship statement

SM and LD designed the study and conducted data collection with participants. Both authors analysed the data, and SM drafted the first draft of the manuscript. SM and LD revised and prepared the final draft of the manuscript for submission.

## Data availability statement

Ethical restriction by the University of Edinburgh Centre for Population Health Sciences Research Ethics Committee prohibits the authors from making the minimal data set publicly available because data contain potentially identifiable information. However, data are available from the corresponding author on reasonable request. Data will be held for five years from February 2017 before being permanently destroyed. Contact: Stephen Malden- stephen.malden@strath.ac.uk.

## References

1. Janssen I, LeBlanc AG. Systematic review of the health benefits of physical activity and fitness in school-aged children and youth. *International journal of behavioral nutrition and physical activity* 2010;7(1):40.
2. Reilly JJ, Armstrong J, Dorosty AR, et al. Early life risk factors for obesity in childhood: cohort study. *Bmj* 2005;330(7504):1357.
3. Gurnani M, Birken C, Hamilton J. Childhood obesity: causes, consequences, and management. *Pediatric Clinics* 2015;62(4):821-40.
4. van Jaarsveld CH, Gulliford MC. Childhood obesity trends from primary care electronic health records in England between 1994 and 2013: population-based cohort study. *Archives of disease in childhood* 2015:archdischild-2014-307151.
5. Reilly JJ, Johnstone A, McNeill G, et al. Results from Scotland's 2016 Report card on physical activity for children and youth. *Journal of physical activity and health* 2016;13(11 Suppl 2):S251-S55.
6. Cooper AR, Goodman A, Page AS, et al. Objectively measured physical activity and sedentary time in youth: the International children's accelerometry database (ICAD). *International journal of behavioral nutrition and physical activity* 2015;12(1):113.
7. Scottish Executive. A curriculum for excellence: Edinburgh: Scottish Executive, 2004.

For peer review only

8. The Daily Mile Foundation. About The Daily Mle Lyndhurst: The Daily Mile Foundation; 2018 [Available from: https://thedailymile.co.uk/about/ accessed 03/08/2018 2018.
9. Chesham RA, Booth JN, Sweeney EL, et al. The Daily Mile makes primary school children more active, less sedentary and improves their fitness and body composition: a quasi-experimental pilot study. *BMC medicine* 2018;16(1):64.
10. Breheny K, Adab P, Passmore S, et al. A cluster randomised controlled trial evaluating the effectiveness and cost-effectiveness of the daily mile on childhood obesity and wellbeing; the Birmingham daily mile protocol. *BMC public health* 2018;18(1):126.
11. Pawson R, Tilley N. An introduction to scientific realist evaluation. 1997
12. Richmond SA, Donaldson A, Macpherson A, et al. Facilitators and Barriers to the Implementation of iSPRINT: A Sport Injury Prevention Program in Junior High Schools. *Clinical journal of sport medicine: official journal of the Canadian Academy of Sport Medicine* 2018
13. Nathan N, Elton B, Babic M, et al. Barriers and facilitators to the implementation of physical activity policies in schools: A systematic review. *Preventive medicine* 2017
14. Ryde GC, Booth JN, Brooks NE, et al. The Daily Mile: What factors are associated with its implementation success? *PloS one* 2018;13(10):e0204988.
15. Executive S. Scottish Index of Multiple Deprivation: Scottish Executive Edinburgh, 2006.
16. Braun V, Clarke V. Using thematic analysis in psychology. *Qualitative research in psychology* 2006;3(2):77-101.
17. Foulds HJ, Bredin SS, Charlesworth SA, et al. Exercise volume and intensity: a dose–response relationship with health benefits. *European journal of applied physiology* 2014;114(8):1563-71.
18. Loprinzi PD, Lee H, Cardinal BJ. Dose response association between physical activity and biological, demographic, and perceptions of health variables. *Obesity facts* 2013;6(4):380-92.
19. Williams PT. Dose-response relationship of physical activity to premature and total all-cause and cardiovascular disease mortality in walkers. *PLoS One* 2013;8(11):e78777.
20. Bailey R. Physical education and sport in schools: A review of benefits and outcomes. *Journal of school health* 2006;76(8):397-401.
21. Kennedy SG, Smith JJ, Hansen V, et al. Implementing Resistance Training in Secondary Schools: An Exploration of Teachers' Perceptions. *Translational Journal of the American College of Sports Medicine* 2018;3(12):85-96.
22. Naylor P-J, Nettlefold L, Race D, et al. Implementation of school based physical activity interventions: a systematic review. *Preventive medicine* 2015;72:95-115.
23. Brustio PR, Moisè P, Marasso D, et al. Feasibility of implementing an outdoor walking break in Italian middle schools. *PloS one* 2018;13(8):e0202091.
24. Howie EK, Pate RR. Physical activity and academic achievement in children: A historical perspective. *Journal of sport and health science* 2012;1(3):160-69.
25. McPherson A, Mackay L, Kunkel J, et al. Physical activity, cognition and academic performance: an analysis of mediating and confounding relationships in primary school children. *BMC public health* 2018;18(1):936.


For peer review only - http://bmjopen.bmj.com/site/about/guidelines.xhtml

26. Rasberry CN, Lee SM, Robin L, et al. The association between school-based physical activity, including physical education, and academic performance: a systematic review of the literature. *Preventive medicine* 2011;52:S10-S20.
27. Dumuid D, Olds T, Martín-Fernández J-A, et al. Academic performance and lifestyle behaviors in Australian school children: a cluster analysis. *Health Education & Behavior* 2017;44(6):918-27.
28. Maher C, Lewis L, Katzmarzyk PT, et al. The associations between physical activity, sedentary behaviour and academic performance. *Journal of science and medicine in sport* 2016;19(12):1004-09.
29. Baker JA, Grant S, Morlock L. The teacher-student relationship as a developmental context for children with internalizing or externalizing behavior problems. *School Psychology Quarterly* 2008;23(1):3.
30. Yunus MM, Osman WSW, Ishak NM. Teacher-student relationship factor affecting motivation and academic achievement in ESL classroom. *Procedia-Social and Behavioral Sciences* 2011;15:2637-41.
31. Scottish Government. Health of Scotland's Population- Obesity Edinburgh: The Scotttish Government; 2017 [Available from: https://www.gov.scot/Topics/Statistics/Browse/Health/TrendObesity accessed 21/05/2018 2018.
32. Scottish Government. A Healthier Future –Scotland's Diet & Healthy Weight Delivery Plan. Edinburgh, 2018.
33. Fairclough SJ, Dumuid D, Mackintosh KA, et al. Adiposity, fitness, health-related quality of life and the reallocation of time between children's school day activity behaviours: A compositional data analysis. *Preventive medicine reports* 2018;11:254-61.

For peer review only

For peer review only



For peer review only - http://bmjopen.bmj.com/site/about/guidelines.xhtml

Appendix 1. Daily Mile Interview Topic Guide

Does the Daily Mile take place at this school? If so, can you give us a brief history of its use at the school? (Were you given any assistance/guidance when setting it up?)

Can you describe a typical day at school for your class- when does the Daily Mile come into play?

What is your understanding of what the Daily Mile actually is? (what does it involve, what are its objectives etc)

When it was first introduced, how did you feel about it? (and have your feelings changed since its implementation?)

Is physical activity a major focus at this school? *(Probe for any physical activity policies that are in place, what does physical education involve/how often is it, is the playground appropriate for physical activity).*

Do you think the children that you teach should be more physically active? *(Are there noticeable issues related to physical activity in the class, obesity, generally unfit etc.).*

In general, how well are the children you teach performing academically? *( are there many children with additional support needs?*

Are there any behavioural issues in the class? *(How well do they listen and follow instructions? Are there any particular times of the day when they are more disruptive/easier or harder to teach?)*

Does their behaviour change following the Daily Mile at all?

Are there any wider issues within the community in which this school is located that you think may hinder/enhance the achievement of children at school? *(Look to discuss the deprivation/affluence of the area, level of engagement with children's learning and development from parents, area safety and crime, provision of adequate facilities. Do these same issues, or others, possibly impact on the children's physical activity levels?).*

Considering all the things we have just discussed, what effect do you think the Daily Mile programme would have on children at this school? *(How would it effect their physical activity levels, would this benefit their overall health? Do you think it would have any effects on their learning or behaviour in class?).*

Thinking practically about what the Daily Mile programme would actually involve, how feasible was it to implement the Daily Mile programme at this school, and keep it running on a daily basis? *(Do you think it is worth sacrificing the classroom time to do it? Do you think other teachers at this school would be in favour of doing the Daily Mile with their class?).*

Were you aware of any challenges that came to light as the Daily Mile was being implemented?

What have been the main barriers for implementing this programme here?

The first school to use the Daily Mile has an all-weather track around the playground for children to use. Do you think the playground at this school is adequate for the Daily Mile to

For peer review only

take place? *(Is there enough space? Would you need to build an all-weather track? What other resources do you think would be required to implement and sustain the programme here?)*

What specific benefits do you think the Daily Mile is having on children at this school? *(Health benefits, academic benefits, behavioural benefits?)*

How do you think parents feel about their children taking part in the Daily Mile, are they aware of it?

What do you think the children think of the Daily Mile?

Considering everything we have discussed, and the specific issues that are present in this area and at this school do you think the introduction of the Daily Mile has been a positive thing overall?

What has been the response from your colleagues?

For peer review only

# Standards for Reporting Qualitative Research (SRQR)*

http://www.equator-network.org/reporting-guidelines/srqr/

**Title and abstract THIS FORM REFERS TO UNMARKED COPY OF MANUSCRIPT**

| | Page/line no(s). |
|---|---|
| **Title** - Concise description of the nature and topic of the study Identifying the study as qualitative or indicating the approach (e.g., ethnography, grounded theory) or data collection methods (e.g., interview, focus group) is recommended | P1 L1-2 |
| **Abstract** - Summary of key elements of the study using the abstract format of the intended publication; typically includes background, purpose, methods, results, and conclusions | P2 L1-25 |

**Introduction**

| | |
|---|---|
| **Problem formulation** - Description and significance of the problem/phenomenon studied; review of relevant theory and empirical work; problem statement | P3 L7-P5 L2 |
| **Purpose or research question** - Purpose of the study and specific objectives or questions | P5 L2-6 |

**Methods**

| | |
|---|---|
| **Qualitative approach and research paradigm** - Qualitative approach (e.g., ethnography, grounded theory, case study, phenomenology, narrative research) and guiding theory if appropriate; identifying the research paradigm (e.g., postpositivist, constructivist/ interpretivist) is also recommended; rationale** | P5 L16-22 |
| **Researcher characteristics and reflexivity** - Researchers' characteristics that may influence the research, including personal attributes, qualifications/experience, relationship with participants, assumptions, and/or presuppositions; potential or actual interaction between researchers' characteristics and the research questions, approach, methods, results, and/or transferability | P7 L6-8 |
| **Context** - Setting/site and salient contextual factors; rationale** | P6 L6-15 |
| **Sampling strategy** - How and why research participants, documents, or events were selected; criteria for deciding when no further sampling was necessary (e.g., sampling saturation); rationale** | P6 L16- P7 L2 |
| **Ethical issues pertaining to human subjects** - Documentation of approval by an appropriate ethics review board and participant consent, or explanation for lack thereof; other confidentiality and data security issues | P6 L1-4 |
| **Data collection methods** - Types of data collected; details of data collection procedures including (as appropriate) start and stop dates of data collection and analysis, iterative process, triangulation of sources/methods, and modification of procedures in response to evolving study findings; rationale** | P7 L5- 15 |

For peer review only

| | |
|---|---|
| **Data collection instruments and technologies** - Description of instruments (e.g., interview guides, questionnaires) and devices (e.g., audio recorders) used for data collection; if/how the instrument(s) changed over the course of the study | P7 L14-15 & Supplementary file |
| **Units of study** - Number and relevant characteristics of participants, documents, or events included in the study; level of participation (could be reported in results) | P6 L14-15; P8 L11-18; Table 1 |
| **Data processing** - Methods for processing data prior to and during analysis, including transcription, data entry, data management and security, verification of data integrity, data coding, and anonymization/de-identification of excerpts | P7 L20- P8 L2 |
| **Data analysis** - Process by which inferences, themes, etc., were identified and developed, including the researchers involved in data analysis; usually references a specific paradigm or approach; rationale** | P7 L19; P8 L3-6 |
| **Techniques to enhance trustworthiness** - Techniques to enhance trustworthiness and credibility of data analysis (e.g., member checking, audit trail, triangulation); rationale** | P7 L20- P8 L3 |

**Results/findings**

| | |
|---|---|
| **Synthesis and interpretation** - Main findings (e.g., interpretations, inferences, and themes); might include development of a theory or model, or integration with prior research or theory | P9 L4- P18 L7 |
| **Links to empirical data** - Evidence (e.g., quotes, field notes, text excerpts, photographs) to substantiate analytic findings | P9 L4 – P18 L7 |

**Discussion**

| | |
|---|---|
| **Integration with prior work, implications, transferability, and contribution(s) to the field** - Short summary of main findings; explanation of how findings and conclusions connect to, support, elaborate on, or challenge conclusions of earlier scholarship; discussion of scope of application/generalizability; identification of unique contribution(s) to scholarship in a discipline or field | P18 L9 – P21 L16 |
| **Limitations** - Trustworthiness and limitations of findings | P21 L17 – P22 L3 |

**Other**

| | |
|---|---|
| **Conflicts of interest** - Potential sources of influence or perceived influence on study conduct and conclusions; how these were managed | P22 L25-26 |
| **Funding** - Sources of funding and other support; role of funders in data collection, interpretation, and reporting | P22 L15 - 20 |

*The authors created the SRQR by searching the literature to identify guidelines, reporting standards, and critical appraisal criteria for qualitative research; reviewing the reference lists of retrieved sources; and contacting experts to gain feedback. The SRQR aims to improve the transparency of all aspects of qualitative research by providing clear standards for reporting qualitative research.



For peer review only - http://bmjopen.bmj.com/site/about/guidelines.xhtml

**The rationale should briefly discuss the justification for choosing that theory, approach, method, or technique rather than other options available, the assumptions and limitations implicit in those choices, and how those choices influence study conclusions and transferability. As appropriate, the rationale for several items might be discussed together.

**Reference:**

O'Brien BC, Harris IB, Beckman TJ, Reed DA, Cook DA. **Standards for reporting qualitative research: a synthesis of recommendations.** *Academic Medicine*, Vol. 89, No. 9 / Sept 2014 DOI: 10.1097/ACM.0000000000000388

For peer review only
